# The Distribution of Pelvic Nodal Metastases in Prostate Cancer Reveals Potential to Advance and Personalize Pelvic Radiotherapy

**DOI:** 10.3389/fonc.2020.590722

**Published:** 2021-01-08

**Authors:** Irina Filimonova, Daniela Schmidt, Sina Mansoorian, Thomas Weissmann, Hadi Siavooshhaghighi, Alexander Cavallaro, Torsten Kuwert, Christoph Bert, Benjamin Frey, Luitpold Valentin Distel, Sebastian Lettmaier, Rainer Fietkau, Florian Putz

**Affiliations:** ^1^ Department of Radiation Oncology, Universitätsklinikum Erlangen, Friedrich-Alexander-Universität Erlangen-Nürnberg, Erlangen, Germany; ^2^ Department of Nuclear Medicine, Universitätsklinikum Erlangen, Friedrich-Alexander-Universität Erlangen-Nürnberg, Erlangen, Germany; ^3^ Institute of Radiology, Universitätsklinikum Erlangen, Friedrich-Alexander-Universität Erlangen-Nürnberg, Erlangen, Germany

**Keywords:** prostate cancer, lymph node metastases, mapping, patterns of recurrence, pelvic radiotherapy

## Abstract

**Background:**

Traditional clinical target volume (CTV) definition for pelvic radiotherapy in prostate cancer consists of large volumes being treated with homogeneous doses without fully utilizing information on the probability of microscopic involvement to guide target volume design and prescription dose distribution.

**Methods:**

We analyzed patterns of nodal involvement in 75 patients that received RT for pelvic and paraaortic lymph node metastases (LNs) from prostate cancer in regard to the new NRG-CTV recommendation. Non-rigid registration-based LN mapping and weighted three-dimensional kernel density estimation were used to visualize the average probability distribution for nodal metastases. As independent approach, the mean relative proportion of LNs observed for each level was determined manually and NRG and non-NRG levels were evaluated for frequency of involvement. Computer-automated distance measurements were used to compare LN distances in individual patients to the spatial proximity of nodal metastases at a cohort level.

**Results:**

34.7% of patients had pelvic LNs outside NRG-consensus, of which perirectal was most common (25.3% of all patients) followed by left common iliac nodes near the left psoas major (6.7%). A substantial portion of patients (13.3%) had nodes at the posterior edge of the NRG obturator level. Observer-independent mapping consistently visualized high-probability hotspots outside NRG-consensus in the perirectal and left common iliac regions. Affected nodes in individual patients occurred in highly significantly closer proximity than at cohort-level (mean distance, 6.6 cm vs. 8.7 cm, p < 0.001).

**Conclusions:**

Based on this analysis, the common iliac level should extend to the left psoas major and obturator levels should extend posteriorly 5 mm beyond the obturator internus. Incomplete coverage by the NRG-consensus was mostly because of perirectal involvement. We introduce three-dimensional kernel density estimation after non-rigid registration-based mapping for the analysis of recurrence data in radiotherapy. This technique provides an estimate of the underlying probability distribution of nodal involvement and may help in addressing institution- or subgroup-specific differences. Nodal metastases in individual patients occurred in highly significantly closer proximity than at a cohort-level, which supports that personalized target volumes could be reduced in size compared to a “one-size-fits-all” approach and is an important basis for further investigation into individualized field designs.

## Introduction

The benefit of elective pelvic radiotherapy in prostate cancer has been repeatedly called into question. The well-known RTOG-9413 trial showed a significant benefit in progression-free survival for prophylactic whole-pelvic radiotherapy with prostate boost and neoadjuvant androgen deprivation therapy (ADT) compared to prostate-only radiotherapy with neoadjuvant ADT (1). However, there was no significant difference between whole-pelvic radiotherapy with neoadjuvant ADT and prostate-only radiotherapy with adjuvant ADT (2). The subsequently conducted randomized GETUG-01 trial also failed to unequivocally prove the superiority of pelvic irradiation in comparison to prostate-only radiotherapy (3). Suboptimal clinical target volume (CTV) definition that missed a substantial proportion of microscopically involved nodes is an important explanation for the lack of clear benefit of pelvic radiotherapy in past randomized trials in the primary setting. At the same time, there is a rising practice of treating nodal oligorecurrent prostate cancer with local ablative therapy (i.e., lymph node dissection or radiotherapy) (4, 5). In case of radiotherapy for oligorecurrent nodal disease, current target volume concepts include involved node stereotactic body radiotherapy (SBRT), involved site SBRT to involved field RT and elective whole pelvic RT without a clear standard having been established yet (4). To clarify the role of elective pelvic radiotherapy in nodal recurrence from prostate cancer, two important multicenter prospective phase II trials including patients with up to five pelvic nodal metastases have been initiated. The STORM trial assesses the benefit of whole pelvic radiotherapy in addition to 6 months of ADT and metastases-directed therapy (salvage lymph node dissection or SBRT) in a randomized fashion (6) and the OLIGOPELVIS-GETUG P07 investigated the impact of pelvic radiotherapy with a simultaneous integrated boost to PET positive nodes plus 6 months of ADT in a single arm phase II design (7).

So far, several attempts have been undertaken to improve CTV design for pelvic radiotherapy in prostate cancer. In 2009, the RTOG-GU radiation oncology specialists published a consensus recommendation on pelvic lymph node volumes for high-risk intact node-negative prostate cancer (8). Furthermore, a variety of studies have investigated nodal recurrence patterns providing valuable insights into the distribution of malignant lymph nodes in prostate cancer (9–14). Several of these studies showed a lack of coverage at important sites of pelvic nodal recurrence especially in the common iliac region above L5/S1 (12–14). For this very reason, the NRG recently put forth an updated international consensus atlas on pelvic nodal volumes for intact node negative as well as node positive and postoperative prostate cancer taking into account critical findings of the last ten years and raising the cranial border to the aortic bifurcation as the most important modification to the previous RTOG consensus recommendation (15). As of yet, patterns of nodal involvement have not been evaluated in regard to this new NRG CTV consensus recommendation.

Moreover, CTV definition for elective pelvic radiotherapy in prostate cancer still largely consists of large volumes being treated with homogeneous doses potentially without fully utilizing information on the probability of microscopic nodal involvement to guide target volume design and prescription dose distribution.

To visualize the average probability distribution for pelvic nodal metastases in prostate cancer, we introduce three-dimensional kernel density estimation after non-rigid registration-based mapping for the analysis of recurrence data in radiotherapy and evaluate results in regard to the newly proposed NRG CTV recommendation. As complementary method, we manually determine the frequency of binary involvement as well as the relative proportion of lymph node metastases for NRG and non-NRG levels and critically analyze coverage of lymph node metastases by the new NRG level definitions. Moreover, we explore preconditions for a potential individualization of target volumes by assessing if nodal metastases in individual patients are more spatially confined than metastases at a cohort-level and by comparing patterns of involvement in patients with and without upfront surgery.

## Materials and Methods

### Ethics

Ethical review and approval was not required for this study on human participants in accordance with the local legislation and institutional requirements (BayKrG Art. 27). Written informed consent to participate in this study was provided by the patients.

### Patient Population

Patients receiving local curative stereotactic radiotherapy of pelvic and/or paraaortic lymph node metastases from prostate cancer in the overall context of a curative or oligometastatic treatment concept between January 2009 to September 2018 were included in this study.

Curative or oligometastatic treatment concept was defined as locally curative treatment (16) of all tumor sites with local curative doses being defined as exceeding an equivalent total dose in 2 Gy fractions (EQD2, alpha/beta = 2) of 50 Gy. Patients who had disseminated disease and those treated with palliative intent were excluded. The treatment indication for radiotherapy of each specific lymph node was based on an interdisciplinary review by experts in diagnostic radiology, nuclear medicine, urology, and radiation oncology after considering the clinical history and all available imaging in each patient case. In general lymph nodes were considered malignant that had significant tracer uptake, a short-axis diameter of ≥1 cm or were enlarging in the context of a rising PSA. Each lymph node included in this study was specifically treated with stereotactic radiotherapy in local ablative intent.

Seventy-five patients treated at our institution fulfilled the abovementioned criteria and were included. Of these 75 cases, 6 patients had paraaortic nodes exclusively and were excluded from analyses investigating the distribution of pelvic nodal metastases, for which the remaining 69 cases were used. Concerning overall tumor stage, 52.0% (39/75) had involved regional lymph nodes only, i.e., cN1 disease located exclusively below the bifurcation of the common iliac arteries. In addition, 36.0% (27/75) had cM1a disease, of which 74.1% (20/27) had paraaortic disease, 51.9% (14/27) had common iliac involvement, 11.1% (3/27) had inguinal metastases, and one patient had a singular mediastinal lymph node in addition to pelvic nodal metastases (3.7%). Only 12.0% (9/75) of patients had cM1b disease, because of additional limited bone metastases. Most patients received conventionally fractionated stereotactic radiotherapy in single doses of 1.8 Gy (97.3%, 73/75). Median EQD2α/β = 2 to involved lymph nodes was 65.0 Gy (range, 53.0–68.4). Lymph node metastases had been identified in most cases by PSMA-PET/CT (42.7%, 32/75) or Choline-PET/CT (30.7%, 23/75), whereas PSMA-SPECT/CT (6.7%, 5/75) and contrast CT (20.0%, 15/75) had been used in the remaining cases (**Table 1**). Importantly, all nodal lesions included in this study went on to receive stereotactic radiotherapy underpinning that the overall clinical certainty in malignant involvement of each lymph node in this analysis was high. There was no significant difference in lymph node region involvement between patients diagnosed with Choline/PSMA-imaging vs. CT alone (**Supplemental Table 1**). In patients who received two series of radiotherapy (17.3%, 13/75), lymph node locations from both treatments were used for analysis.

**Table 1:** Patients’ characteristics.

Patient characteristic	Total cohort (N = 75)
**D’Amico risk group at first diagnosis, n (%)**	
High risk	60 (80.0%)
Intermediate risk	14 (18.7%)
Low risk	1 (1.3%)
**Gleason score at first diagnosis, n (%)**	
5	2 (2.7%)
6	6 (8.0%)
7	30 (40.0%)
8	18 (24.0%)
9	15 (20.0%)
10	4 (5.3%)
**iPSA at first diagnosis, ng/ml**	
Median (range)	12.7 (3.3–431)
	
**T stage at first diagnosis, n (%)**	
T1a	2 (2.7%)
T1b	2 (2.7%)
T1c	3 (4.0%)
T2a	4 (5.3%)
T2b	4 (5.3%)
T2c	21 (28.0%)
T3a	19 (25.3%)
T3b	18 (24.0%)
T4	2 (2.7%)
**Primary treatment, n (%)**	
Prior radical prostatectomy	55 (73.3%)
Prior antiandrogenic therapy	18 (24.0%)
Prior radiotherapy	10 (13.3%)
prostatic fossa only	8 (10.7%)
prostatic fossa and elective RT of pelvic lymph node levels	2 (2.7%)
**R-Status (resected patients only)**	
R0	34 (61.8%)
R1	11 (20.0%)
Unknown	10 (18.2%)
**Number of initially resected nodes (resected patients only)**	
Median (range)	16 (3–55)
**Initial pN stage (resected patients only)**	
pN0	42 (76.4%)
pN1	13 (23.6%)
**Time interval between primary treatment** **and RT for nodal recurrence, years**	
Median (IQR)	4.8 (1.4–9.1)
**Age at start of RT for nodal metastases, years**	
Median (range)	70 (43–85)
**M stage at start of RT for nodal metastases, n (%)**	
cM0	39 (52.0%)
cM1a	27 (36.0%)
cM1b	9 (12.0%)
**Imaging technique for detection of nodal metastases, n (%)**	
PSMA-PET/CT	32 (42.7%)
Choline-PET/CT	23 (30.7%)
PSMA-SPECT/CT (99mTc-MIP-1404)	5 (6.7%)
Contrast CT	15 (20.0%)

PSMA-PET, prostate specific membrane antigen positron emission tomography; CT, computed tomography; RT, Radiotherapy; IQR, Interquartile range.

### Mapping Analysis

In all patients with pelvic nodal metastases (n = 69), verified GTV segmentations were exported from the treatment planning system (Iplan, Brainlab Feldkirchen Germany) and imported into 3DSlicer (v.4.10.2) (17). Geometric centers of every lymph node segmentation were calculated and in every patient, the Euclidean distance between all pelvic lymph node center coordinates was computed using the function cdist of the Python library SciPy (18) (401 distances in total) before mapping.

A patient CT dataset that best represented the average anatomy of the cohort served as common template and mapping target. Non-rigid registration was performed with the 3DSlicer SlicerRT Plastimatch B-spline deformable registration module (17, 19, 20). After a first rigid registration step (subsampling 2 × 2 × 1, maximum of 100 iterations), a 3-stage B-Spline deformable registration (stage 1: subsampling 2,2,1, grid 25 mm, regularization 0.01, landmark penalty 0.005, maximum iterations 100, stage 2: subsampling 2,2,1, grid 10 mm, regularization 0.01, landmark penalty 0.005, maximum iterations 100, stage 3: subsampling 1,1,1, grid 2 mm, regularization 0.01, landmark penalty 0.005, maximum iterations 100) with Mean Squared Error as cost function empirically provided the best results and was used in all cases. The resulting deformation vector fields were used to map the lymph node center locations from each patient into the common template anatomy. The quality of the non-rigid registration and the resulting mapping locations were reviewed by a radiation oncologist and accepted in all cases. After mapping, the Euclidean distances between all 210 mapped pelvic lymph node center locations were computed using SciPy (18) (21,945 unique distances in total) and compared to the lymph node distances obtained *via* intra-patient measurements (401 distances).

Kernel density estimation was applied to convert the mapped lymph node center locations into an estimate of the underlying average probability distribution for metastatic lymph node involvement. Kernel density estimation is a widely used and accepted statistical technique to estimate the underlying probability density function from a limited set of observations (21). Three-dimensional kernel density estimation based on the mapped lymph node center locations was performed using the Python library KDEpy (22) (rectangular kernel, bandwidth 1.25 cm, p-norm 2), which results in a spherical representation of mapped nodal locations. To avoid bias toward patients with a high number of lymph nodes, weighting was applied, so that each patient contributed equally to the estimate irrespective of the number of positive nodes. To facilitate visualization the resulting spatial distribution was smoothened using the ITK recursive gaussian filter (sigma 2.5) (23). Three-dimensional renderings and a CT atlas of the average distribution of pelvic nodal metastases were created with 3DSlicer (17).

### Expert-Based Assessment and Statistical Analysis

Independent from the mapping technique, the patterns of lymph node involvement were analyzed by expert-based assessment as a complementary method. For every patient dataset, a radiation oncologist evaluated the number of metastatic lymph nodes in all pelvic levels as well as in the paraaortic region. The results were reviewed by a second radiation oncologist and confirmed in all cases. For the definition of lymph node levels, the NRG consensus recommendations were used except for perirectal and inguinal regions, where the RTOG consensus for anorectal cancer was employed as these regions are not included in the NRG consensus for prostate cancer (15, 24).

The frequency of binary involvement was calculated for every region at a patient-level. Differences between patients with and without prior surgery were evaluated using Fisher’s exact test with Bonferroni adjustment for multiple testing.

In addition to binary involvement, the varying extent of metastatic involvement was quantified for pelvic lymph node levels. To avoid bias toward patients with a high number of lymph nodes, calculations were not carried out at the level of individual lymph nodes. Instead, the relative contribution of each pelvic lymph node level to the total amount of positive nodes was first determined in each patient and this patient-level metric was subsequently evaluated for the whole population. The relative proportion of lymph nodes observed for each level was normalized by the level volume to identify possible hotspot regions. Analysis was limited to NRG and perirectal lymph node regions. Level volumes were determined *via* segmentation in the template dataset. In addition, we statistically tested if the mean relative proportion of lymph node metastases observed for each level was significantly different from the value theoretically expected by a homogeneous distribution of nodal metastases. A bootstrapped one-sample T-test and a bootstrapped 95% CI of the mean (BCa-based bootstrapping, 10,000 bootstrap samples) was used as normality could not be assumed. Time-to-event metrics were estimated using the Kaplan-Meier method and calculated from the start of stereotactic radiotherapy to local progression of irradiated nodal metastases (local control), local or distant progression (freedom from progression) or death from prostate cancer (disease-specific survival) with patients being censored at last follow-up or death, respectively. Median follow-up of this cohort was 58.9 months. Statistics were calculated using IBM SPSS 21. Graphs were created using GraphPad Prism 7 and SPSS.

## Results

**Figure 1:**
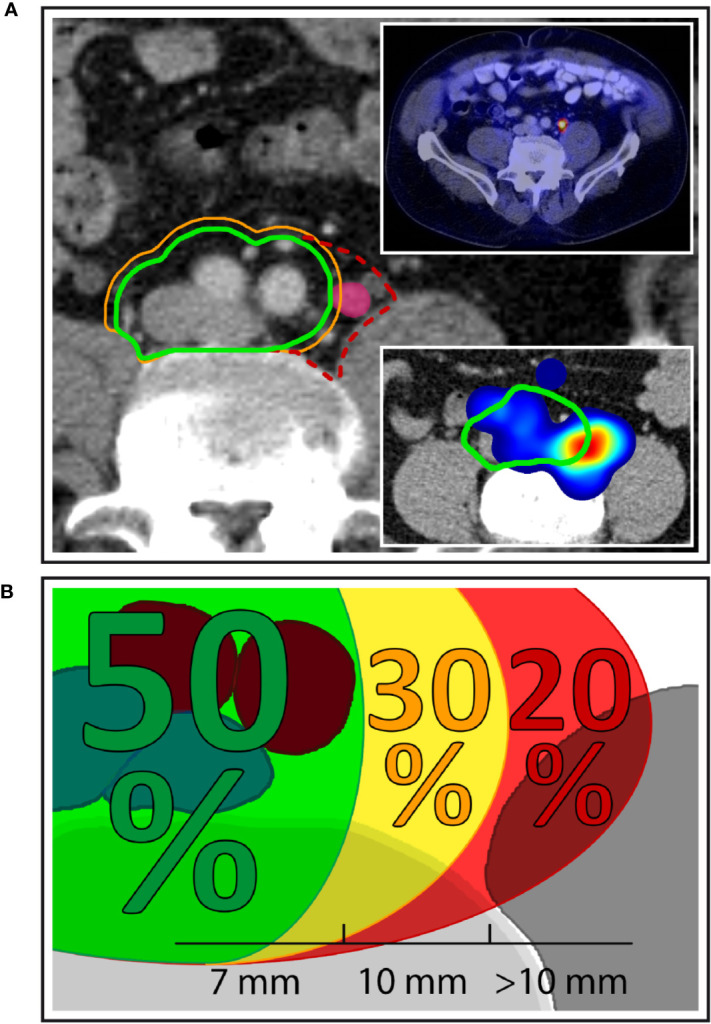
Left common iliac nodal metastases outside NRG consensus CTV. **(A)** Exemplary patient case with a left common iliac nodal metastasis outside the NRG CTV recommendation using margin options of 7 mm (green contour) and 10 mm (orange contour), respectively. Dashed red contour: proposed extension of the CTV to the medial surface of the left major psoas muscle. Top right inset: PSMA-PET/CT of the same patient demonstrating PSMA uptake. Bottom right inset: Corresponding anatomic location in the independent mapping analysis showing a high-probability hotspot that extends beyond the left boundary of the NRG CTV recommendation. **(B)** Overall, 50.0% of patients with left common iliac nodal involvement had all common iliac metastases covered using a margin of 7 mm around the vessels. A 10 mm margin encompassed all nodal metastases in an additional 30.0% of patients, while 20.0% of patients with left common iliac metastases had lymph node metastases that extended even beyond the 10-mm margin.

**Figure 2:**
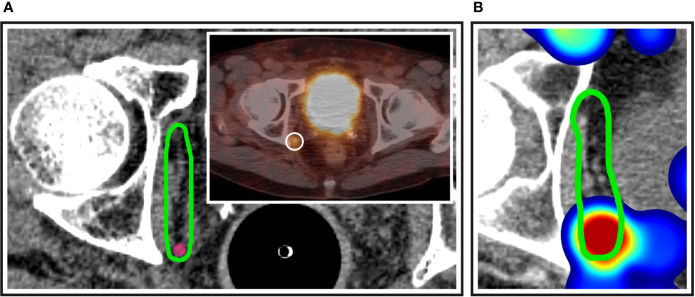
Obturator nodes at the posterior edge of the NRG consensus CTV. **(A)** Exemplary patient case with a PSMA-avid nodal metastasis (inset) right at the posterior edge of the NRG obturator level. **(B)** Corresponding anatomic location in the independent mapping analysis showing a high-probability hotspot near the posterior edge of the NRG CTV recommendation.

In total, 92.0% (69/75) of patients had pelvic lymph node involvement, while in 8.0% (6/75), only paraaortic nodes were present. In addition, 24.0% of patients (18/75) suffered from both pelvic and paraaortic lymph node metastases. The median number of involved lymph node regions per patient was 2 (range, 1–9 and interquartile range, 1–2), differentiating left and right levels, respectively. Pelvic lymph node involvement was strictly unilateral in 76.8% of patients (53/69), whereas 23.2% (16/69) had metastatic pelvic nodes in left as well as right lymph node levels. The median number of malignant pelvic nodes in each patient was 2 (range, 1–11) and 36.2% (25/69) had only one metastatic pelvic lymph node. In patients with at least two positive pelvic nodes, the median of the maximum intra-patient lymph node distance was 7.8 cm, the 75th%ile was 11.0 cm and the 95th%ile was 17.0 cm. The external iliac lymph node region was most frequently involved (37.3%, 28/75) followed by the paraaortic (32.0%, 24/75), internal iliac and perirectal (25.3%, 19/75 each), common iliac (22.7%, 17/75), obturator (20.0%, 15/75), and the presacral region (10.7%, 8/75). The inguinal and prevesical lymph node region, not included in the new NRG CTV recommendation, each only harbored metastatic lymph nodes in 4.0% of patients (3/75). One of the most important additions in the new NRG consensus CTV is the inclusion of common iliac nodal levels above L5/S1 that had not been part of the previous RTOG-GU consensus volume. 18.7% of patients (14/75) had positive nodes in the common iliac region that were located above L5/S1 and thus outside the previous RTOG-GU consensus recommendation. However, when carefully analyzing the location of left common iliac metastases, we observed that a fraction of these lymph nodes also was located outside the new NRG CTV recommendation in between the left boundary of the NRG consensus CTV and the medial surface of the left psoas major muscle (**Figure 1**). In addition, 50% (5/10) of patients with left common iliac metastases had nodal metastases outside the NRG consensus recommendation corresponding to 6.7% (5/75) of all patients, when using a standard margin of 7 mm around the vessels. The NRG consensus recommendation gives the option to increase this margin to 10 mm particularly anterior to vessels, if clinically indicated (15). However, even when using this more generous margin, still 20% (2/10) of patients with left common iliac involvement or 2.7% (2/75) of all patients had common iliac metastases outside the NRG consensus CTV volume, respectively. Importantly, all common iliac nodal metastases would have been covered if the left CTV boundary had extended to the medial surface of the left psoas major muscle (**Figure 1**). An additional important observation was made regarding obturator nodes. Despite no obturator node was indisputably located outside the NRG consensus CTV, 10 patients had obturator metastases located right near the posterior edge of the NRG consensus CTV corresponding to 66.7% (10/15) of patients with obturator level involvement and 13.3% (10/75) of all patients (**Figure 2**). In total, excluding paraaortic involvement, 34.7% of patients (26/75) had metastatic lymph nodes not included in the new NRG consensus, of which perirectal was the most frequent (25.3%, 19/75 patients) (**Table 2**).

Frequency of lymph node involvement.

**Table 2A:** 

Lymph node region n (%)	Total cohort (N = 75)
External iliac	28 (37.3%)
**Paraaortic**	**24 (32.0%)**
Internal iliac	19 (25.3%)
**Perirectal**	**19 (25.3%)**
Common iliac	17 (22.7%)
**Left common iliac outside NRG (7/10 mm)**	**5 (6.7%)/2 (2.7%)**
Obturator	15 (20.0%)
Presacral	8 (10.7%)
**Inguinal**	**3 (4.0%)**
**Prevesical**	**3 (4.0%)**

Regions indicated in bold are not part of the NRG consensus recommendation.

**Table 2B:** Frequency of lymph node involvement with discrimination of left and right lymph node groups.

Lymph node region n (%)	Left	Right
External iliac	19 (25.3%)	15 (20.0%)
Paraaortic	20 (26.7%)	19 (25.3%)
Internal iliac	15 (20.0%)	7 (9.3%)
Perirectal	11 (14.7%)	8 (10.7%)
Common iliac	10 (13.3%)	11 (14.7%)
Obturator	5 (6.7%)	10 (13.3%)
Inguinal	1 (1.3%)	3 (4.0%)
Prevesical	2 (2.7%)	1 (1.3%)

**Figure 3:**
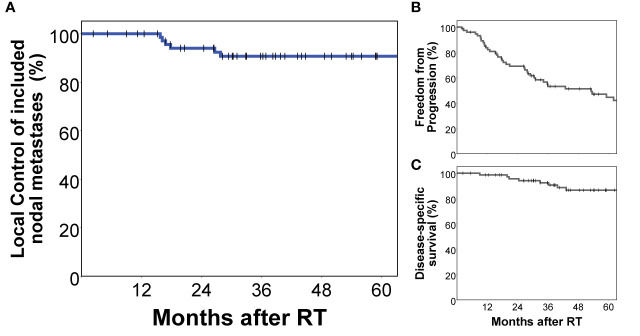
Treatment outcomes of the analyzed cohort. All nodal metastases included in this analysis had been specifically treated with stereotactic radiotherapy in local ablative intent. Local control of included nodal metastases was 90.7% at 5 years **(A)**. Freedom from local and distant progression **(B)** as well as prostate cancer-specific survival **(C)** of this cohort are also shown.

Interestingly, right external iliac involvement was significantly reduced in patients with previous prostatectomy and pelvic lymph node dissection compared to patients without prior surgery (10.9% vs. 45.0%, p = 0.002, p adjusted for multiple testing 0.022, **Table 3**). All nodal metastases included in this study received stereotactic radiotherapy in local ablative intent. Local control of nodal metastases following stereotactic radiotherapy was 90.7% at 5 years. Disease-specific survival of this cohort was 86.6% and freedom from local and distant progression was 44.5% at 5 years post radiotherapy (**Figure 3**).

**Table 3:** Differences in lymph node region involvement in patients with and without prior prostatectomy with pelvic lymph node dissection.

Lymph Node Region	Resection part of primary treatment		
	No	Yes	P
Common iliac, left	5.0%	16.4%	0.272
Common iliac, right	25.0%	10.9%	0.150
Internal iliac, left	25.0%	18.2%	0.526
Internal iliac, right	10.0%	9.1%	1.000
External iliac, left	35.0%	21.8%	0.368
**External iliac, right**	**45.0%**	**10.9%**	**0.002**
Obturator, left	5.0%	7.3%	1.000
Obturator, right	10.0%	14.5%	1.000
Perirectal, left	10.0%	16.4%	0.717
Perirectal, right	10.0%	10.9%	1.000
Presacral	0.0%	14.5%	0.100

P, Fishers exact test.

The observed difference in right external iliac involvement remained significant after Bonferroni correction for multiple testing (p = 0.022).

### Lymph Node Mapping and Kernel Density Estimation

**Figure 4:**
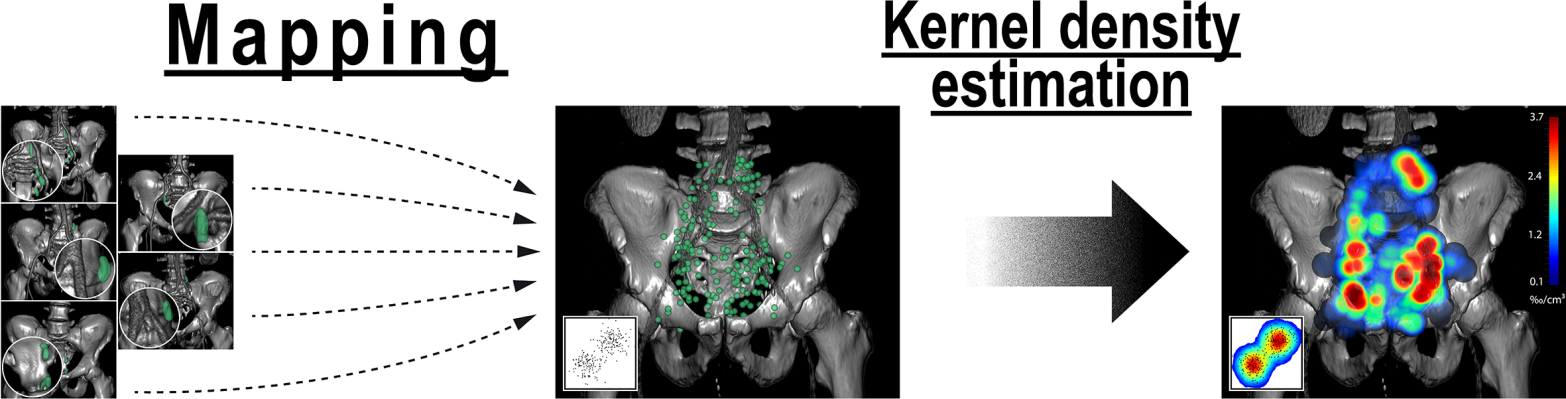
Analysis of the average probability distribution for nodal metastases by observer-independent mapping and three-dimensional kernel density estimation. First, lymph node center locations from all patients were mapped into a common template CT (left). Weighted three-dimensional kernel density estimation was then applied to convert the mapped lymph node center locations into an estimate of the underlying average probability distribution for lymph node metastases (right). Weighting was applied so that each patient case contributed equally to the estimate irrespective of the number of positive nodes. Kernel density estimation is a widely used and accepted statistical technique to estimate the underlying probability density function from a limited set of observations that is most commonly applied for one- and two-dimensional data (inset). Note: Kernel density estimation helps in visually identifying regions with high density of nodal involvement without the need to restrict the analysis to predetermined level boundaries.

The geometric centers of pelvic nodes from all patients were mapped into a common template CT using an observer-independent non-rigid registration-based mapping technique (**Figure 4**). After mapping, distances between all lymph nodes from all patients were calculated (210 lymph nodes, 21945 unique distances) and compared to the lymph node distances obtained *via* intra-patient measurements (401 distances). The mean distance between involved pelvic lymph nodes was highly significantly smaller in individual patients than at a cohort-level (6.6 cm vs. 8.7 cm, p < 0.001), i.e., metastatic nodes were significantly closer. This was equally true, if distances between lymph nodes in individual patients were measured after being mapped into the common anatomy of the template dataset (6.1 cm vs. 8.7 cm, p < 0.001) showing that the increase in mean lymph node distance at a cohort-level was not artificially introduced by the mapping procedure.

**Figure 5:**
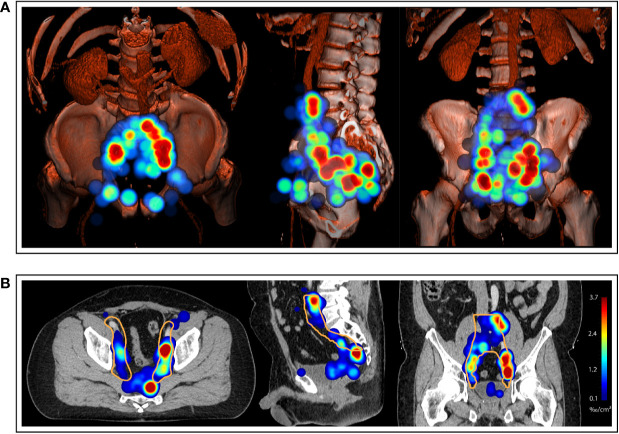
**(A)** Three-dimensional renderings visualize the estimated three-dimensional probability density function of pelvic nodal metastases (maximum intensity projection) for the whole cohort in a common reference CT (composite with shading). **(B)** CT atlas reconstructions (Solid orange contour: Clinical target volume defined according to the recent NRG recommendation). Left column: superior/axial view, middle column: left-side view/sagittal reconstruction, right column: anterior view/coronal reconstruction. Weighting was applied so that each patient case contributed equally to the estimate irrespective of the number of positive nodes.

**Figure 6:**
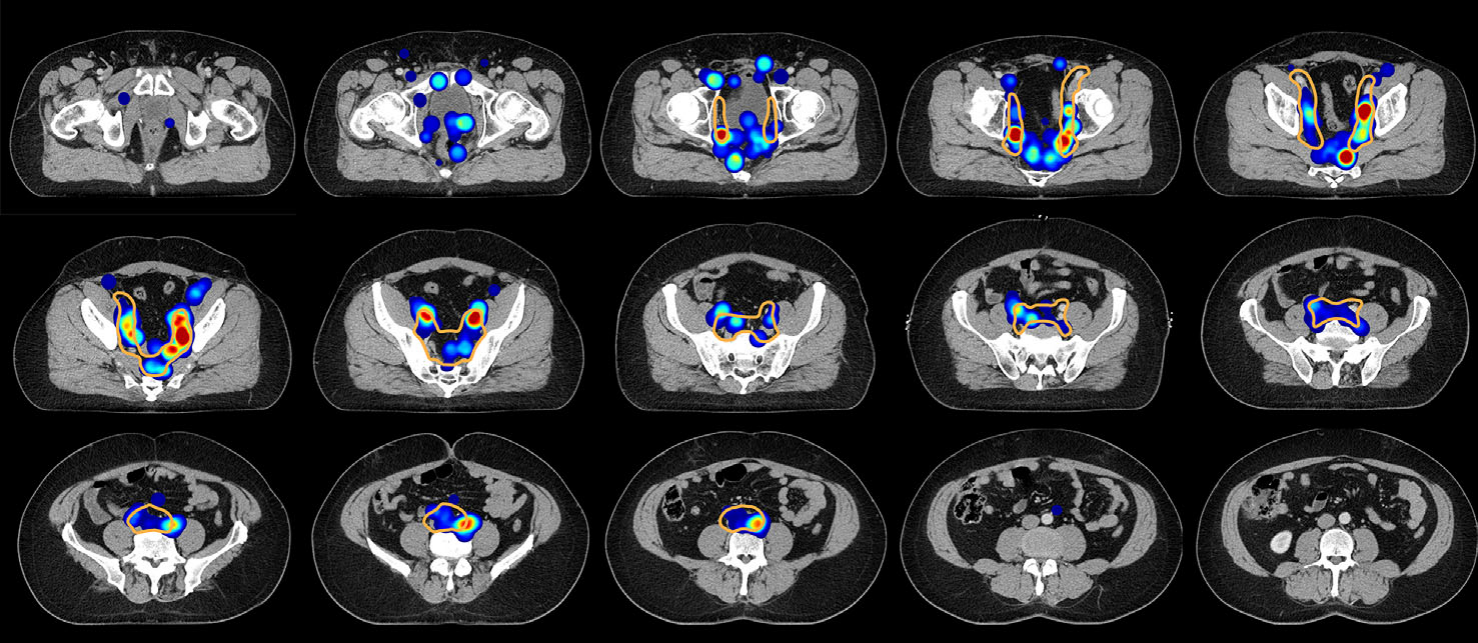
Axial slices of the created CT atlas visualize the estimated three-dimensional probability density function of pelvic nodal metastases for the whole cohort in a common reference CT. Weighting was applied so that each patient case contributed equally to the estimate irrespective of the amount of positive nodes. Slice spacing is 15 mm. Solid orange contour: Clinical target volume defined according to the recent NRG recommendation.

For the mapping analysis, weighted three-dimensional kernel density estimation was used to assess the underlying average probability distribution of pelvic lymph node metastases. The CT atlas and three-dimensional renderings illustrate the estimated three-dimensional probability density function of pelvic nodal metastases in ‰ per cm³ for the whole cohort in a common reference CT with weighting being applied so that each patient contributes equally to the estimate irrespective of the amount of positive nodes. Hotspots of lymph node involvement outside NRG consensus are visualized especially in the perirectal region (**Figures 5** and **6**, **Supplemental Video S1**). Consistent with the manual assessment, the independent mapping analysis also revealed a high-probability hotspot extending beyond the NRG consensus volume in the left common iliac region and high risk for malignant nodes near the posterior edge of the NRG obturator level (**Figures 1** and **2**).

### Expert-Based Assessment

As an independent approach to the mapping technique, the relative distribution of lymph node metastases was determined by a radiation oncologist for every patient.

To avoid bias toward patients with many positive nodes, the relative contribution of a lymph node level to the total amount of positive nodes was first determined in every individual patient and this patient-level metric was subsequently evaluated for the whole population.

**Figure 7:**
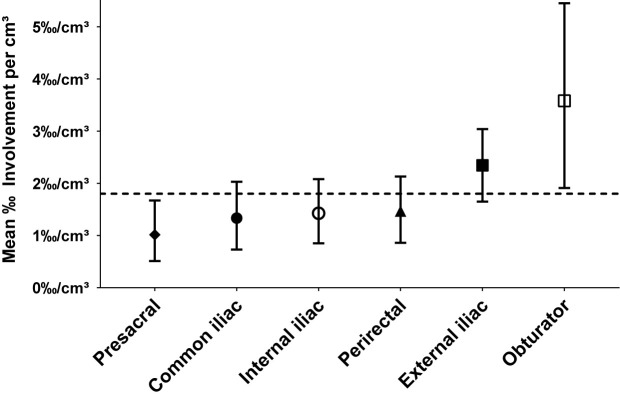
Mean relative proportion of lymph node metastases per cm³ in typical pelvic lymph node levels (n = 66). Error bars indicate 95% CI obtained by BCa-based bootstrapping using 10,000 bootstrap samples. The dashed reference line indicates the mean relative proportion expected by a homogeneous spatial distribution of lymph node metastases.

The mean proportion of metastatic lymph nodes per level volume was highest for the obturator levels (3.58‰/cm³), followed by external iliac (2.34‰/cm³), perirectal (1.47‰/cm³), internal (1.43‰/cm³), and common iliac (1.33‰/cm³). The proportion of involved lymph nodes for the presacral level was lowest (mean 1.01‰/cm³), significantly lower than expected by a homogeneous spatial distribution of lymph node metastases (p = 0.047). When differentiating by side, the right obturator (4.01‰/cm³), left obturator (3.14‰/cm³), left external iliac (2.60‰/cm³), and left internal iliac (2.08‰/cm³) levels showed the highest mean relative lymph node involvement per cm³ (**Figure 7**).

## Discussion

Renewed interest in the distribution of pelvic nodal metastases has been sparked by recent evidence that pelvic nodal recurrence represents an important site of treatment failure in prostate cancer (25). Suboptimal CTV design is an important explanation for the lack of clear benefit of pelvic radiotherapy in past randomized trials for intact prostate cancer (2, 3). On the other hand, there is rising use of local ablative approaches in the treatment of nodal oligorecurrent prostate cancer with radiotherapy target volumes varying from involved-node SBRT over involved site and involved field approaches to elective pelvic radiotherapy (4, 5).

A variety of studies have investigated patterns of recurrence after radical prostatectomy and/or radiotherapy, some of them in relation to the previous RTOG-GU consensus recommendation, and have provided important information for the optimization of radiotherapy field design and the extent of surgical dissection (9–14). Because of this new evidence, an updated NRG international consensus atlas on pelvic lymph node volumes for pelvic radiotherapy in prostate cancer was recently published taking into account critical findings of the last ten years of published research. To obtain a new consensus for CTV design in pelvic radiotherapy 18 international experts had contoured the nodal CTV for an intact node-negative, intact node-positive as well as a postoperative prostate cancer case after a systematic literature review. Regions of controversy were subsequently identified by evaluating nodal CTVs from all experts and a consensus was reached. The inclusion of all common iliac nodes up to the aortic bifurcation in the new NRG CTV certainly is the most significant modification over the previous RTOG-GU recommendation (15). To the best of our knowledge, the present study is the first to analyze the coverage of pelvic nodal metastases according to the new NRG consensus recommendation.

We found that 34.7% of patients harbored metastatic pelvic nodes outside the recently published NRG consensus recommendation for prostate cancer. Perirectal lymph nodes were most frequent (25.3% of all patients) followed by left common iliac nodes in between the left boundary of the NRG consensus CTV and the medial surface of the left psoas major muscle (6.7%), whereas inguinal and prevesical involvement only occurred in 4.0% of patients each. Recommendations for an expansion of the previous RTOG-GU consensus CTV definition had been derived from multiple studies. Spratt et al. found increasing coverage when raising the cranial CTV border with a coverage of 33.3% at S1/S2, 41.7% at L5/S1, and 93.4% at L4/L5 (13). In a large recent study of 82 patients, De Bruycker et al. obtained compatible findings and found that by raising the cranial field border from L5/S1 to the aortic bifurcation an additional 22.8% (36/158) out of all pelvic and paraaortic lesions analyzed in the study could be covered (14). The cranial border also was a crucial factor in our analysis to completely cover all common iliac metastases. These results are very important for the interpretation of past randomized trials investigating the benefit of pelvic radiotherapy: The partially positive RTOG 9413 used L5/S1 as cranial border (1), whereas the negative GETUG-01 study used S1/S2 (3). Considering the results from the aforementioned trials and those from our own investigation, it seems evident why it was not possible to achieve a positive effect with a S1/S2 cranial border setting (3).

As an elevation of the cranial field border up to the aortic bifurcation is the most significant addition in the new NRG consensus CTV, it was a particularly important finding that left common iliac nodes were not completely covered by the new NRG CTV in a substantial portion of patients in the present study. In addition, 50.0% (5/10) of patients with left common iliac involvement had metastases in between the left boundary of the NRG consensus CTV and the medial surface of the psoas major muscle using the recommended standard margin of 7 mm around the vessels. Based on this observation, we recommend completely covering the space from the common iliac vessels to the left psoas major muscle when delineating common iliac nodal levels using the medial surface of the psoas major as left-side boundary.

An additional noteworthy observation was made in regard to obturator nodes. Despite none of them was undoubtedly outside the NRG consensus CTV, the majority of patients (66.7%, 10/15) with obturator involvement had lymph node metastases right at the posterior boundary of the NRG obturator level, i.e., near the posterior edge of the obturator internus muscle and were endangered of being missed by a restricted application of the NRG contouring guidelines.

However, by far the most common reason for incomplete NRG coverage of nodal metastases was perirectal involvement. Consistent with our finding that the perirectal level was the most frequently involved region outside the NRG consensus, the largest share of lesions missed by the previous RTOG-GU CTV recommendation (10/20) was also located in the perirectal region in a PSMA-PET–based patterns of failure analysis by Schiller and coauthors (12). While NRG GU experts thoroughly discussed the inclusion of perirectal levels especially for T4 tumors, the majority of experts had opted against routinely including these lymph nodes in view of the corresponding increase in treatment volume (15). As the perirectal region has been a frequent site of nodal recurrence in multiple studies (12, 14, 26), it would be highly beneficial to identify reliable predictive factors for perirectal involvement to include perirectal nodes in high-risk patients but to avoid unnecessary toxicity in others.

It is important to note that care has to be taken when interpreting the results from patterns of involvement studies. First, the diagnostic methods used for the detection of nodal metastases may have an important influence, as the major role of PSMA-PET imaging on radiotherapy treatment planning has already been shown (12, 27). In the cohort by Spratt et al., lymph node metastases were detected in 92.3% *via* CT/MRI imaging and in only 7.7% *via* PET-CT (13), which could explain differences in lymph node involvement. In our cohort, PSMA-PET/CT and Choline-PET/CT were the most common imaging methods for detecting lymph node metastases (73.3%, 55/75), but some were detected by PSMA-SPECT/CT or contrast CT alone. However, the fact that all lymph node metastases were specifically treated with stereotactic radiotherapy after interdisciplinary review underpins that the overall clinical certainty of the malignant involvement of included nodes was high in our series.

The definition of lymphatic regions in patterns of recurrence studies generally is governed by the overall study setting, be it centered on surgical, diagnostic or radiotherapeutic objectives. In the atlas of patterns of spread of prostate cancer, Barbosa et al. describe a diagnostic view of lymph node regions (9). Then again, Spratt et al. analyzed patterns of nodal failure according to Morón et al. using 34 abdomino-pelvic stations (13, 28). These circumstances can lead to possible misinterpretation of study results. In the present analysis, NRG consensus lymph node level definitions were used wherever possible to allow for optimal interpretation of results in regard to radiotherapy field design (15). For the same reason, if metastatic nodal involvement extended beyond the recent NRG consensus, e.g., to inguinal and perirectal regions, we chose RTOG compartment definitions from other cancer types. Concerning NRG consensus levels, the external and internal iliac levels were most commonly involved in our series (37.3% and 25.3% of patients, respectively), whereas the presacral region was only involved in 10.7% of patients.

Furthermore, previous treatments and therefore patient selection might also determine patterns of involvement. Comparing our results to the literature, our observed distribution of pelvic nodal metastases has evident similarities with studies consisting mostly of patients with upfront surgery. Thus, our findings show similarities to the study of Calais et al., which included patients with biochemical recurrence after prostatectomy, as well as to the analysis of McClinton et al., in which the majority of patients had prior resection (64.8% in the study by McClinton et al., 73.3% in our series) (10, 11). In the study from Calais et al., PSMA-PET/CT mapping was performed in a cohort of 270 patients with biochemical recurrence after radical prostatectomy and a PSA < 1.0 ng/ml. In the 83 patients with positive nodes on PSMA-PET/CT, the most frequent site of nodal recurrence was external iliac (45.8%), followed by internal iliac (32.5%) and obturator level nodes (22.9%) (10). Our finding of significantly different nodal involvement patterns in patients with and without upfront surgery generally supports this notion that prior surgery could influence the distribution of lymph node recurrences. Whereas our observation of reduced right external iliac involvement in patients with prior prostatectomy and pelvic lymph node dissection must not be generalized, it could reflect a preferential dissection of these lymph node levels by referring surgeons in the present series. Interestingly, Meijer et al. had observed a high occurrence of aberrant nodal metastases, especially perirectal involvement, with magnetic resonance lymphography following radical prostatectomy also affirming the hypothesis that prior surgery may influence nodal recurrence patterns. Interestingly, 43% of patients in the study by Meijer et al. harbored perirectal nodes followed by 36% with metastatic lymph nodes in the left common and left internal iliac region each (29).

Finally, De Bruycker et al. analyzed nodal recurrence patterns in patients with biochemical failure and ≤ 5 nodal metastases on choline-PET/CT with most patients (82.9%) having initially been treated with radical prostatectomy or a combination of radical prostatectomy and postoperative radiation (14). Most nodal metastases in the work by De Bruycker et al. were located in the external iliac region (28.5%), followed by common iliac (24.1%) and paraaortic (13.3%) metastases, whereas only 6.3% of positive nodes were located in the perirectal area (14).

It is important to note, that De Bruycker et al. analyzed coverage of nodal metastases not only for radiotherapeutic but also for surgical approaches. The results show that the superextended salvage lymph node dissection included more lesions compared to the new extended, limited or standard lymph node dissection but had a comparable coverage to elective pelvic radiotherapy using the top of L4 as superior border. However, lymph node metastases in at least 31% of patients, especially all perirectal lesions, would still have been missed by all the surgical dissection templates as well as the extended radiotherapy field design (14).

Urological series that analyze the topographic distribution of positive lymph nodes in primary lymphadenectomy are also an important source of evidence for nodal dissemination patterns especially for patients presenting with intact prostate cancer. In 74 patients with primary node negative prostate cancer, Joniau et al. analyzed the patterns of lymph node involvement obtained *via* a sentinel node procedure and superextended lymphadenectomy. Most, histologically proven, lymph node metastases were located in the internal iliac region (35%), followed by the external iliac (26%) and obturator region (25%) (30).

Optimal target volume design is also a pressing question in the context of nodal oligorecurrent prostate cancer, as there is a rising practice of treating nodal oligorecurrent prostate cancer with salvage lymph node dissection and/or radiotherapy (5). In retrospective case series and phase II trials, these metastasis-directed therapies have shown promising progression-free survival with limited toxicity (5, 28). In case of radiotherapy for oligorecurrent nodal disease, current target volume concepts vary from involved node SBRT, involved site SBRT to involved field RT and elective whole pelvic RT without a clear standard having been established yet (4). As most patients treated with SBRT for nodal recurrence alone, however, relapse in adjacent lymph node regions within 24 months there is a good rationale for the exploration of more generous radiotherapy target volume concepts (6). In a systematic review of the mostly retrospective literature, Achard et al. found improved progression-free survival with elective nodal radiotherapy compared to involved-node SBRT in nodal oligorecurrent prostate cancer (4). Following the urgent need for better evidence, two important prospective trials have been initiated to evaluate the oncologic efficacy and toxicity of pelvic radiotherapy in nodal oligorecurrent prostate cancer. De Bruycker et al. are assessing the benefit of whole pelvic radiotherapy in addition to ADT plus salvage lymph node dissection or SBRT for pelvic nodal oligorecurrence (≤ 5 nodes) from prostate cancer in the randomized phase II STORM trial (6). The second trial, the OLIGOPELVIS-GETUG P07 investigated the impact of salvage pelvic radiation therapy plus ADT with a simultaneous integrated boost to a maximum of 5 pelvic metastases in a single-arm phase II design (7).

Notably, both prospective oligorecurrence trials employ the RTOG-GU consensus CTV but with an elevated cranial field border (L4/L5 in the STORM and aortic bifurcation in the OLIGOPELVIS-GETUG P07 trial) (6, 7). The main concern with an elevated cranial field border is of course a higher toxicity rate. However, the already completed OLIGOPELVIS-GETUG P07 trial was able to show a limited toxicity rate (10% urinary and 2% intestinal ≥ grade 2 toxicity at 2 years). Most importantly demonstrating a 2-year progression-free survival of 77.6% the single-arm phase II OLIGOPELVIS-GETUG P07 trial also met its prespecified primary endpoint providing a clear efficacy signal for whole pelvic radiotherapy in the oligorecurrent setting (28). The results of the randomized STORM study are expected for 2024 and are eagerly awaited (6). We performed distance measurements between metastatic pelvic lymph nodes in individual patients and found a median maximum intra-patient lymph node distance of 7.8 cm with a 75th%ile of 11.0 cm and a 95th%ile of 17.0 cm suggesting the need for larger field sizes to adequately cover microscopic nodal involvement in a majority of patients.

As microscopically involved lymph nodes cannot be detected *via* imaging, they only exist as probabilities at the time of pelvic radiotherapy. Aside from analyzing the frequency of binary involvement for NRG lymph node regions and beyond, an important aim of this study was to provide an estimate for these probabilities to gain further insights into how to optimize radiotherapy field design and prescription dose distribution.

We did this using two independent but complementary methods. First, we used lymph node mapping based on an observer-independent non-rigid registration technique and kernel density estimation to create a voxel-level visualization of the average distribution of nodal metastases. While mapping studies in prostate cancer have employed observer-dependent mapping or even rigid registration techniques (10, 12, 13), this is the first study to use an observer-independent non-rigid registration-based mapping technique for the analysis of nodal involvement in prostate cancer. Three-dimensional kernel density estimation was employed to convert the mapped lymph node locations into an estimate of the underlying three-dimensional probability density function for metastatic lymph node involvement. Weighting was applied so that each patient contributed equally to the estimate irrespective of the number of positive nodes. To the best of our knowledge, this is the first study to introduce three-dimensional kernel density estimation for the analysis of recurrence data. Kernel density estimation is a widely used and accepted statistical technique to estimate the underlying probability density function from a limited set of observations (21). This non-parametric method is widely used in the one-dimensional and two-dimensional setting, e.g., to analyze geographic patterns of disease incidence from a limited number of observations in two dimensional maps (31, 32). By improving visualization and analysis of recurrence data, kernel density estimation could ultimately aid in deriving meaningful insights for optimal CTV design. As these methods can be easily automated, software solutions could be developed that allow fast and convenient patterns of failure analyses in routine clinical practice. This could be especially important, as the average distribution of nodal involvement may vary for different radiotherapy treatment centers, because of local surgical preferences, among others.

The developed mapping and kernel density estimation technique also provided valuable insights in the present study by visualizing the average probability of nodal involvement in reference to the consensus-based NRG CTV recommendation and helped identify regions of suboptimal coverage in the common iliac and obturator region.

Frequently, patterns of recurrence analyses are performed at the level of individual lymph nodes without correction, which might introduce a bias toward the patterns of metastatic spread of patients with many metastatic nodes (11, 12, 14, 33). In our series, lymph node metastases in individual patients occurred in more spatially confined clusters than at a cohort-level, which suggests that the probability distribution of nodal metastases might vary between individual patients. Consequently, the overall pattern obtained when assessing individual lesions without correction will be biased toward patients with an above-average amount of metastatic nodes and misrepresents the pattern for an average patient case. In addition, tumors with a large amount of lymph node metastases may have an unusual underlying biology, which might also affect the pattern of lymph node involvement. In the present analysis, we assessed the spatially varying quantitative extent of metastatic involvement in such a way that every patient case was weighted equally irrespective of the number of involved nodes.

As an independent approach to the observer-independent mapping analysis, the varying quantitative extent of metastatic involvement was quantified manually for all pelvic lymph node levels. We found that the proportion of metastatic lymph nodes was highest for the obturator and external iliac levels (mean of 3.58‰/cm³ and 2.34‰/cm³, respectively) followed by the perirectal level (mean 1.47‰/cm³), whereas the mean proportion of lymph node metastases was lowest for the presacral level (1.01‰/cm³), significantly lower than expected by a homogeneous distribution of nodal metastases. Our findings collectively support the notion that lymph node metastases are not distributed uniformly across pelvic levels but that the average probability of nodal involvement varies regionally. Based on our findings, obturator and external iliac levels could serve as candidate levels for dose escalation, while the presacral level could be considered for sparing. The perirectal level also was an important region of involvement in this study suggesting that inclusion of perirectal lymph nodes could be beneficial for a subgroup of patients.

These are important findings that warrant confirmation as a validated description of the regionally varying average probability of microscopic lymph node involvement could not only inform the setting of boundaries of radiotherapy target volumes but could even be translated into a non-uniform prescription dose distribution to allow for local dose-escalation and -sparing.

Moreover, the unprecedented finding that nodal metastases were highly significantly more spatially confined in individual patients than at a cohort-level indicates that pelvic radiotherapy in prostate cancer could be substantially improved, if it could be individualized on an individual patient basis compared to a “one-size-fits-all” approach. While advances in machine learning could enable highly-individualized target volumes in the future, it is equally important to compare patterns of involvement for major patient subgroups and to assess institution-specific differences in the location of high-risk regions. Observer-independent mapping and three-dimensional kernel density estimation could be helpful tools in these investigations toward more personalized radiotherapy field designs.

### Limitations

Limitations of this study are the sample size that precluded some subgroup analyses. While the retrospective nature of this study increased heterogeneity in included patients, strict prospective patient selection could also have introduced biases and misrepresented nodal involvement for an average patient case in clinical practice. The majority but not all included patients were diagnosed with PSMA- or Choline-PET/CT. However, the fact that all nodes did receive radiotherapy underpins that the clinical certainty in the malignant involvement of included nodes was high.

## Conclusion

This is the first study to investigate patterns of nodal involvement in regard to the new NRG CTV consensus recommendation. 34.7% of patients had pelvic nodal involvement outside NRG CTV consensus in this study, of which perirectal was the most common. An important observation was that the NRG consensus CTV missed left common iliac nodes in a considerable fraction of patients that could easily be covered by extending the CTV to the medial surface of the left major psoas muscle. In addition, as obturator nodes in many patients were located near the posterior edge of the NRG consensus CTV, we recommend to consider extending the obturator level 5 mm beyond the posterior edge of the obturator internus muscle.

Furthermore, we introduced three-dimensional kernel density estimation after non-rigid registration-based mapping for the analysis of recurrence data in radiotherapy. This technique provides an estimate of the underlying probability distribution of nodal involvement, can be fully automated and thus may help in addressing institution- or subgroup-specific differences. In this study, the developed mapping technique provided valuable insights for analysis of the new NRG consensus CTV. We propose considering analyses that weight every patient case equally in patterns of recurrence studies, as unadjusted analyses at the level of individual lesions could introduce bias toward patients with many metastases. In our study, the relative proportion of involved nodes was highest for the obturator and external iliac levels while it was lowest for the presacral level making these candidate regions for dose-escalation and sparing, respectively. Prior surgery and local surgical preferences could influence the distribution of nodal recurrences and warrant further study. Nodal metastases in individual patients occurred in highly significantly closer proximity than at a cohort-level, which supports that personalized target volumes could be reduced in size compared to a “one-size-fits-all” approach and is an important basis for further investigation into individualized field designs.

## Data Availability Statement

The raw data supporting the conclusions of this article will be made available by the authors, without undue reservation.

## Ethics Statement

Ethical review and approval was not required for the study on human participants in accordance with the local legislation and institutional requirements. The patients/participants provided their written informed consent to participate in this study.

## Author Contributions

IF conducted the manual experiments and contributed with the development of the mapping and kernel density estimation technique and the other employed analyses. FP developed the mapping and kernel density estimation technique, developed the other employed analyses, was responsible for the statistical analyses, and conducted the 3DSlicer and Python based analyses including writing the necessary program code. IF, FP, DS, SM, TW, HS, AC, TK, CB, BF, LD, SL, and RF contributed to writing the manuscript. IF, FP, SL, CB, and RF conceptualized this work. FP, AC, TK, CB, and RF provided the resources. DS and TK provided nuclear medicine and AC provided radiologic expertise for conducting this work. All authors contributed to the article and approved the submitted version.

## Funding

FP was supported by a grant from the Interdisciplinary Center for Clinical Research (IZKF) Erlangen: rotation program for physician scientists (https://www.izkf.med.fau.de/).

## Conflict of Interest

CB reports grants from Siemens Healthineers, outside the submitted work. RF reports grants and personal fees from Merck Serono, grants and personal fees from Astra Zenica, grants and personal fees from MSD, grants and personal fees from Novocure, personal fees from Brainlab, personal fees from Fresenius Kabi, personal fees from Bristol Meyers Sqibb, and personal fees from Sennewald GmbH, outside the submitted work. FP reports grants and personal fees from Siemens Healthineers, outside the submitted work.

The remaining authors declare that the research was conducted in the absence of any commercial or financial relationships that could be construed as a potential conflict of interest.
