# Supplementary material for: The Distribution of Pelvic Nodal Metastases in Prostate Cancer Reveals Potential to Advance and Personalize Pelvic Radiotherapy
Source: Front Oncol. 2021 Jan 8;10:590722. doi: 10.3389/fonc.2020.590722 (PMC7820617; doi:10.3389/fonc.2020.590722)
Supplement: Supplementary file 1 [file Table_1.docx]

Supplementary Material

# Supplementary Table S1

Differences in lymph node region involvement in patients with and without nuclear medicine-based imaging

|  | Nuclear medicine-based imaging | | |
| --- | --- | --- | --- |
| **Lymph Node Region** | **Yes** | **No** | **P** |
| Common iliac, left | 15.0% | 6.7% | 0.676 |
| Common iliac, right | 15.0% | 13.3% | 1.000 |
| Internal iliac, left | 18.3% | 26.7% | 0.483 |
| Internal iliac, right | 6.7% | 20.0% | 0.138 |
| External iliac, left | 23.3% | 33.3% | 0.510 |
| External iliac, right | 18.3% | 26.7% | 0.483 |
| Obturator, left | 8.3% | 0.0% | 0.576 |
| Obturator, right | 11.7% | 20.0% | 0.408 |
| Perirectal, left | 15.0% | 13.3% | 1.000 |
| Perirectal, right | 11.7% | 6.7% | 1.000 |
| Presacral | 13.3% | 0.0% | 0.345 |
| Common iliac, any | 25.0% | 13.3% | 0.496 |
| Internal iliac, any | 21.7% | 40.0% | 0.186 |
| External iliac, any | 35.0% | 46.7% | 0.552 |
| Obturator, any | 20.0% | 20.0% | 1.000 |
| Perirectal, any | 26.7% | 20.0% | 0.747 |

P = Fishers exact test
